# Evaluating inter-rater reliability of indicators to assess performance of medicines management in health facilities in Uganda

**DOI:** 10.1186/s40545-018-0137-y

**Published:** 2018-05-03

**Authors:** Belinda Blick, Stella Nakabugo, Laura F. Garabedian, Morries Seru, Birna Trap

**Affiliations:** 1 Management Sciences for Health, Plot 15, Princess Anne Drive, Bugolobi, P.O. Box 71419, Kampala, Uganda; 2 Harvard Pilgrim Health Care Institute, 401 Park Drive Suite 401,, Boston, MA 02215 USA; 3 Ministry of Health, Pharmacy, Division, Lourdel Road, Wandegeya, Kampala Uganda

**Keywords:** Data reproducibility, Inter-rater reliability, IRR, Medicines management indicators, Data quality audit, Performance assessment quality, Simple indicators, Complex indicators

## Abstract

**Background:**

To build capacity in medicines management, the Uganda Ministry of Health introduced a nationwide supervision, performance assessment and recognition strategy (SPARS) in 2012. Medicines management supervisors (MMS) assess performance using 25 indicators to identify problems, focus supervision, and monitor improvement in medicines stock and storage management, ordering and reporting, and prescribing and dispensing. Although the indicators are well-recognized and used internationally, little was known about the reliability of these indicators. An initial assessment of inter-rater reliability (IRR), which measures agreement among raters (i.e., MMS), showed poor IRR; subsequently, we implemented efforts to improve IRR. The aim of this study was to assess IRR for SPARS indicators at two subsequent time points to determine whether IRR increased following efforts to improve reproducibility.

**Methods:**

IRR was assessed in 2011 and again after efforts to improve IRR in 2012 and 2013. Efforts included targeted training, providing detailed guidelines and job aids, and refining indicator definitions and response categories. In the assessments, teams of three MMS measured 24 SPARS indicators in 26 facilities. We calculated IRR as a team agreement score (i.e., percent of the MMS teams in which all three MMS had the same score). Two sample tests for proportions were used to compare IRR scores for each indicator, domain, and overall for the initial assessment and the following two assessments. We also compared the IRR scores for indicators classified as simple (binary) versus complex (multi-component). Logistic regression was used to identify supervisor group characteristics associated with domain-specific and overall IRR scores.

**Results:**

Initially only five (21%) indicators had acceptable reproducibility, defined as an IRR score ≥ 75%. At the initial assessment, prescribing quality indicators had the lowest and stock management indicators had the highest IRR. By the third IRR assessment, 12 (50%) indicators had acceptable reproducibility, and the overall IRR score improved from 57% to 72%. The IRR of simple indicators was consistently higher than that of complex indicators in the three assessment periods. We found no correlation between IRR scores and MMS experience or professional background.

**Conclusions:**

Assessments of indicator reproducibility are needed to improve IRR. Using simple indicators is recommended.

**Electronic supplementary material:**

The online version of this article (10.1186/s40545-018-0137-y) contains supplementary material, which is available to authorized users.

## Background

Like many other developing countries, Uganda faces serious financial and human resource constraints in the health sector [1]. For every 100,000 citizens, there are only 1.6 pharmacists; only 8% of public sector pharmacist posts and 61% of pharmacy technician posts were filled in 2013/14 [2]. Because of the inadequate number of pharmacy professionals, health workers from different cadres are often called on to perform tasks related to medicines management in their facilities despite their lack of appropriate training [2]. This results in problems that negatively affect service delivery (e.g., drug stock-outs) and waste limited resources through inappropriate management. Surveys in public sector facilities depict a challenging environment; in 2010, less than 10% of facilities had all six vital tracer medicines available, no facilities had correctly filled stock cards, and only 1% of facilities provided the correct treatment for a simple cough and cold [3, 4]. The Ministry of Health’s traditional approach of providing short training courses to address the knowledge and skills gap in medicines management has not produced significant or sustainable improvements [3, 5].

Using multi-pronged approaches to build capacity and change behaviors has proven to be more effective compared to single approaches [6, 7], and in 2012, following pilot results, Uganda’s Ministry of Health introduced a new national multi-pronged approach to build health worker capacity—the supervision, performance assessment and recognition strategy (SPARS). SPARS combines supervision—in the form of “supportive supervision”—with performance assessment to identify problem areas, guide supervision, and track improvements. A recognition strategy rewards good performance. Selected district health facility staff members trained as medicines management supervisors (MMS) are tasked to build capacity at health facilities by implementing SPARS [8].

The MMS assess medicines management performance in five SPARS domains: 1) stock management, 2) storage management, 3) dispensing, 4) prescribing, and 5) ordering and reporting quality. They use a standardized indicator-based assessment tool that includes 25 indicators. The SPARS indicators in the five domains are listed in Table 1. As part of their training, the MMS receive an orientation on how to use the tool that includes the indicators’ background and purpose; data sources and data collection method; and indicator calculation, analysis, and interpretation. MMS gather indicator data during each supervisory visit by interviewing exiting patients, observing health workers’ practices, and auditing records; the data gathering method used depends on the indicator. They receive netbooks and internet modems to facilitate data entry, analysis, and reporting [8].

**Table 1:** List of the 25 SPARS indicators by the five domains

Dispensing quality	Description
1. Dispensing time*	Measures active/interactive dispensing time for 6 patients. Excludes any interruptions and time spent on communication unrelated to the patient condition or medication
2. Packaging material	Measures availability of appropriate dispensing materials like envelopes for solid dosage forms and bottles for liquid dosage forms. Paper cones and reused bottles were considered inappropriate
3. Dispensing equipment	Measures availability of dispensing equipment for both liquid and solid dosage forms like spoon, spatula, measuring cylinder, tablet counting tray to ensure that tablets are not counted by bare hands
4. Services available at the dispensing area	Measures access to privacy, chairs and benches, hand washing facilities at the facility and drinking water for patients within the dispensing area
5. Patient care*	Measures discrepancy between dispensed and prescribed medications and adequacy of information provided to patients at dispensing (dose, frequency, duration, why to take and other information required for adherence to medication)
6. Labeling*	Measures adequacy of information on the label (medicines name, strength, quantity, date, dose, name of patient and facility)
7. Rationing of antibiotics	Measures the practice of rationing antibiotics when in short supply. Antibiotic quantities prescribed and dispensed for 5 patients are compared to establish rationing, using amoxicillin and cotrimoxazole as examples
Prescribing quality	Description
8. Correct use of prescription recording system	Measures appropriate recording of 10 prescriptions dispensed (date, OPD/IP number, diagnosis, medicine and prescribers’ name, quantity of medicine prescribed and dispensed)
9. Rational prescribing*	This standard World Health Organization indicator measures appropriate prescribing medicines in 20 prescriptions, assessing average number of medicines prescribed per patient, percent of products prescribed as generics, percent of prescriptions containing antibiotics, percent of prescriptions containing injections, and percent of prescriptions with diagnosis recorded
10. Adherence to STG for diarrhoea	Measures adherence to STG for non-bloody diarrhoea treatment. Appropriate treatment is ORS and zinc only
11. Adherence to STG for common cough/cold (simple respiratory tract infection)	Measures adherence to STG for cough/ cold. Appropriate treatment is optional antipyretic/analgesic without use of antibiotics
12. Adherence to STG for malaria	Measures adherence to STG for treatment of non-complicated malaria. Appropriate treatment with antimalarials only should always follow a positive test
Stock management	Description
13. Availability of stock card	Measures availability of stock cards based on basket of 15 stock items
14. Correct filling of stock card	Measures correct filling of stock cards (medicines name, strength, dosage form, average monthly consumption, special storage conditions)
15. Does physical count agree with recorded stock card balance	Measures whether stock balance according to stock card agrees with counted physical stock
16. Stock book** correctly used	Measures correct use of stock book (all column information is appropriately filled and calculated, including average monthly consumption and quantity to order)
Storage management	Description
17. Cleanliness of the pharmacy	Measures cleanliness of the dispensary and main store (floor, wall, shelves and medicines are checked)
18. Hygiene of the pharmacy	Measures availability, functionality, and hygiene of designated sanitary facilities for dispensary staff (toilet, toilet paper, hand washing and soap).
19. System for storage of medicines and supplies	Measures if medicines in the facility are stored on shelves/cupboards in an appropriate and systematic manner and the shelves are labelled
20. Storage conditions (main store)	Measures appropriate physical storage conditions and steps taken to assure quality and safety of medicines in storage (sign of pest, protection from light, temperature monitoring and regulation, roof condition, storage space, lockable storage, fire safety equipment, cold storage, separate storing of medicines/vaccines appropriately in refrigerator, recording temperature in refrigerator)
21. Storage practices of medicines in pharmacy (stores & dispensary)	Measures adherence to good storage practices (incorrect storage on the floor, expired items recorded and stored separately, FEFO, opened bottles labelled with opening date, and lids on all containers)
Ordering and reporting	Description
22. Reorder level calculation	Measures ability of the facility to correctly calculate reorder quantity
23. Timeliness of order & distribution***	Measures adherence to order and delivery schedules (only applicable for higher level facilities)
24. Accuracy of HMIS reports	Measures if the health facility staff update the HMIS 105 report with accurate information on medicines availability during the previous month from stock management records. Stock card and HMIS 105 information are compared for consistency for a basket of 6 EMHS.
25. Filing	Measures appropriate filing of previous orders, delivery notes and discrepancy reports

Notes: OPD=outpatient department; IP=inpatient; ORS=oral rehydration solution; STG=standard treatment guidelines; FEFO=First expiry first out; HMIS=Health Management Information System; EMHS=essential medicines and health supplies

*World Health Organization indicators or sub indicators; **Stock book summarize in one line the monthly transactions from the stock card ***Excluded from assessment

The SPARS facility assessment data are reported upward to district health offices and national program managers through a computerized national pharmaceutical management information system. High-performing health facilities, district health officers, and MMS are recognized with rewards such as mobile phone air and modem time, T-shirts, tea, calendars, soap, branded wall clocks, and mugs. Since SPARS started piloting at the end of 2010, MMS have submitted more than 12,000 SPARS facility reports. Using medicines management performance data, managers can identify problems more quickly and make data-informed decisions. Facilities supervised to date have documented improvements in medicines management [9].

Many indicators have been developed to assess various aspects of medicines management and pharmaceutical sector performance, and country programs use indicators to identify problems and monitor progress [10, 11]. Too often, however, programs apply well-known and well-tested indicators, such as the World Health Organization rational drug use indicators, without assessing the quality of data or indicator reproducibility or investing the time and effort needed to assure that the standards of data collection and interpretation are met [10, 12, 13].

To ensure data quality and reproducibility of indicator-based tools, it is important that the data collector has enough training and practice to develop a sufficient understanding of what the indicators are measuring and how to use them [10, 14, 15]. Data reliability is a critical issue, especially when data are used to make program and policy decisions. Suggested strategies to improve data quality include inter-rater reliability (IRR) assessments that measure agreement among independent raters about their rating of a characteristic or behavior plus efforts to improve IRR, if it is insufficient [16–18].

From the outset, the Ministry of Health’s Pharmacy Department prioritized efforts to assure data reliability, because the SPARS data would be used to make programmatic and policy decisions for the sector. The Ministry of Health uses the SPARS facility scores to implement a performance and certification program; therefore, it is critically important that the SPARS scores are reliable and independent of the rater (MMS) assessing the performance.

As part of the development of the SPARS facility assessment tool, we carried out a small exploratory study in July 2011 to assess IRR of the SPARS indicators. The low IRR scores in this initial assessment led to targeted efforts to increase IRR. The research question for the current study was to assess IRR for SPARS indicators and to evaluate if targeted interventions improved inter-rater reliability. The study objectives were to assess IRR for 24 SPARS indicators collected by well-trained MMS at three different time points and to examine whether IRR increased after efforts to improve reproducibility, which included revisions of the tool, development of guidelines, and additional MMS training.

## Methods

### MMS selection

To assess IRR, we used teams composed of three MMS each. The MMS who participated in the IRR assessments were randomly selected from the pool of all active MMS at the time of the study. In the first assessment, 54 MMS were active, which increased to 151 by the second assessment, and 224 by the third assessment. The active MMS were grouped into “experienced” or “less experienced” based on the number of SPARS facility assessments that the MMS had completed prior to the IRR assessment. At the initial assessment, the threshold for MMS to be considered experienced was seven or more SPARS facility assessments; this threshold increased to ≥12 visits in the following two assessments because MMS had more time to complete additional visits and thereby gain experience. In all three assessments, the MMS were randomly selected from the two different experience groups to make up rater teams with two experienced MMS and one less-experienced MMS. The initial assessment included only two rater teams, which increased to 10 rater teams per assessment in the second and third assessments. A total of 66 MMS participated.

### The three IRR assessments

The initial IRR assessment in July 2011 was conducted to determine baseline IRR. The second (March–June 2012) and third (February–April 2013) IRR assessments were specifically designed and carried out to evaluate whether the revision of the tool, new guidelines, and additional MMS training had contributed to improved IRR scores. In the initial assessment, the two MMS teams each assessed three facilities (six facilities total). In the two following assessments, the 10 teams each assessed two facilities, and each facility was assessed by two teams, totaling 26 facilities in all three assessments (Table 2).

**Table 2:** Summary of efforts to improve reproducibility and IRR assessments

Efforts flow	Timing	Number of MMS rater teams (total # of MMS)	# IRR facility assessments by each team	Total # of IRR assessments	# facilities by level of care (High/Low)
IRR assessment: 1	Jul 2011	2 (6)	3	6	2 High, 4 Low
Effort: 1	Jan 2012	All MMS receive effort 1			
IRR assessment: 2	Mar-Jun 2012	10 (30)	2	20	10 Low
Effort 2	Sep 2012	All MMS receive effort 2			
IRR assessment: 3	Feb-Apr 2013	10 (30)	2	20	10 Low
Total	Jul 2011-Apr 2013	22 (66)	Not applicable	46	2 High, 24 Low

We selected the facilities purposefully to consider accessibility from the facilities the MMS had planned to supervise. The initial baseline assessment included two higher level facilities. The next two assessments only included lower level facilities, which are higher in number (93%) and only have one medicine store, making it faster to collect data for stock management indicators.

### Data collection

When the MMS team visited a facility, each team member independently collected the data needed and scored the SPARS indicators using the standardized SPARS data collection tool and method (Additional files 1 and 2). One SPARS indicator (#23) was excluded from the assessment because it only applied to higher-level facilities. A study investigator oversaw each assessment to ensure that the MMS did not communicate with each other during the SPARS data collection. The investigator did not influence or interfere in the data collection. We recorded the characteristics of MMS teams to explore possible relationships of IRR scores and rater team composition, including gender, profession, and experience.

The MMS did not receive additional training or orientation on the purpose of the study prior to the IRR assessment. For SPARS indicators that required record sampling (e.g., outpatient register records and dispensing log), the investigator pre-selected the records to be used by all team members. Patient exit interviews were conducted with the same patient; one MMS conducted the interview, but all MMS recorded their assessments independently. MMS assessed dispensing time for the same patients, but individually. In the stores, MMS observed the storage conditions and collected stock management and ordering and reporting information individually as per their basic MMS training.

Once each MMS independently completed the assessment at the health facility using the SPARS tool, the study investigator collected the tools and compiled the scores from each MMS for each indicator in an Excel spreadsheet. For the purpose of the IRR analysis, we classified the 24 SPARS indicators into two groups based on their complexity (Table 3). *Simple* indicators are those that require binary yes or no answers, and *complex* indicators are composites with sub-indicators that require sampling and calculations.

**Table 3:** Classification of SPARS indicators by complexity

Simple indicators (13/24)	Complex indicators (11/24)
2. Packaging material 3. Dispensing equipment 4. Services available at dispensing areas 7. Rationing of antibiotics 13. Availability of stock card 14. Correct filling of stock card 16. Stock book correctly used 17. Pharmacy cleanliness 18. Pharmacy hygiene 19. Storage system for medicines and supplies 20. Storage conditions 21. Storage practices in store and dispensary 25. Filing	1. Dispensing time 5. Patient care 6. Labelling 8. Correct use of prescription recording system 9. Rational prescribing 10. Adherence to STG for diarrhea 11. Adherence to STG for cough/cold 12. Adherence to STG for malaria 15. Physical count agrees with stock card balance 22. Reorder level calculation 24. Accuracy of HMIS reports

### Scoring

We looked at the SPARS scores to assess agreement across the three-person team (i.e., team agreement score) to calculate an IRR score for each indicator. We used a slightly different approach to assess team agreement score depending on the type of indicator:

- For yes or no responses, the team agreement score was 100% if all the three MMS agreed; otherwise, it was 0% [18].
- In a continuous scale, a team agreement score of 100% was given if all three MMS had a SPARS score within +/− 10% of the median value for the group. If not, the team agreement score was 0%.
- For the indicator *dispensing time*, a team agreement score of 100% was given if all three MMS assessed the average dispensing time for the patients within +/− 15 s of the median value for the group. If not, the IRR score was 0%.

For indicators that had sub-questions or sub-indicators, the team agreement score was separately assessed for each sub-question and then averaged across the sub-questions for that indicator. We calculated the average percentage agreement across all MMS teams to measure the IRR for an indicator (i.e., the proportion of teams that scored 100%). An illustration of the IRR score calculation for indicators, sub-indicators, and domains is provided (Additional file 3).

Inter-rater reliability was deemed “acceptable” if the IRR score was ≥75%, following a rule of thumb for acceptable reliability [19]. IRR scores between 50% and < 75% were considered to be moderately acceptable and those < 50% were considered to be unacceptable in this analysis.

### Statistical analysis

For each of the three IRR assessment periods, we calculated the average IRR for each of the 24 indicators, indicator category (simple or complex), the five SPARS domains and overall SPARS score and compared the IRR scores of the three IRR assessment periods using a two-sample test for proportions.

To determine whether there was an association between MMS group characteristics and SPARS reliability, we used logistic regression to estimate the odds ratio and the 95% CI associated with having a score of ≥75% for each SPARS domain by MMS team composition type. The MMS team composition characteristics that we assessed were gender (i.e., number of males on the team), profession, and experience based on the average number of SPARS visits carried out by the team prior to the IRR assessment. All these analyses were conducted using STATA, Version 13 and Excel 2007.

### Efforts to improve measurement reliability

Prior to making SPARS a national strategy, we piloted the performance assessment tool with simple instructions over 12 months and made several adjustments during that time. In 2010 the tool was finalized for national rollout and became the basis for the MMS two-week classroom training and five-day practical training.

In July 2011, we carried out the first exploratory IRR assessment, and although the sample was small, the findings led to the development of training programs to increase IRR scores (Table 2). We then applied interventions that are proven to be effective in increasing reliability [20]. To increase IRR by reducing errors in measurement procedures and interpretation, we developed guidelines, refined indicator wording and definitions in the tool to increase clarity, and re-trained MMS in the problematic indicators identified by the IRR assessments (< 75% IRR score).

Starting in January 2012, detailed data collection guidelines were developed to supplement the simple instructions. We now included in the data collection tool descriptive information on each indicator that had been highlighted during training, including information on the background, purpose, and operational definitions of indicators, and guidelines on methods to collect, analyze, and interpret data. Some indicator response categories were simplified to make MMS assessment more straightforward; for example, *cleanliness of dispensary and main store* was revised from the possible response categories—*very clean/tidy (score 1), acceptable clean/tidy (score 0.5),* and *not clean/untidy (score 0),* to only two categories: *clean/tidy (score 1)* and *not clean/untidy (score 0).* For other indicators, MMS were given examples of what they should look for in their assessment, for example for *presence of pests in the store,* we advised them to check for wasp nests, cobwebs on the ceilings, termites along the walls, and small droppings of bats or rats. These efforts were followed by the second IRR assessment in March–June 2012 (Table 2).

**Fig. 1:**
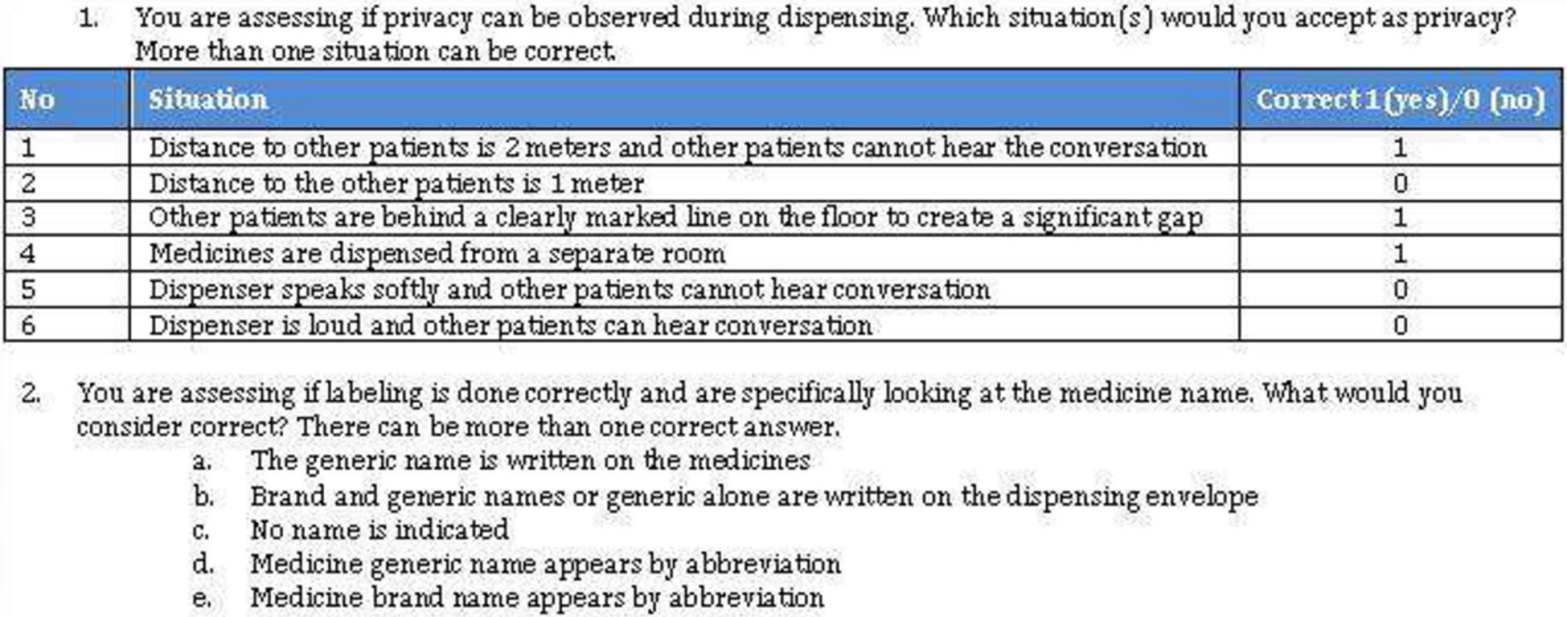
Example of SPARS indicator exercise from the MMS training

In September 2012, we issued a second revision of the tool and guidelines that further clarified challenging indicators. The final SPARS data collection guidelines are provided in Additional file 2. After the introduction of the revised tool, all MMS attended a targeted two-day training course that focused on the problematic indicators and other frequent errors. We focused heavily on the complex indicators that involve several steps, including multiple calculations, to produce the SPARS indicator score and the correct use of zero and “not applicable” and how to address blank fields. To test individual MMS’ understanding of the focus indicators, we incorporated practical exercises using multiple choice questions. An example of such an exercise is given in Fig. 1. The group would discuss the answers to achieve a common understanding.

We realized that MMS’ calculation skills differed considerably because they come from diverse health-related professional backgrounds, and that we needed to consider this in the training design. Consequently, we added more test examples to give them ample practice. We refined the standard operating procedures for SPARS data management to clarify information on tracking facility visits, data cleaning, data security, and reporting. The September 2012 revision and training were followed by the third and final IRR assessment in February–April 2013 (Table 2). To reduce errors, we also shifted from a manual tool to an electronic tool that automatically calculates the scores for some of the indicators. However, the electronic SPARS data collection was not rolled out until December 2013, after the study period.

### Ethical considerations

This study evaluated IRR of medicines management data as part of the national capacity-building strategy SPARS carried out by MMS under the Ministry of Health, Uganda. The study did not involve patients, human or personal health data, human tissue, or animals. Therefore, the study did not require ethical approval or a waiver. All observations and data collection were conducted with the permission of Ministry of Health, the District Health Officers, the facility in-charges, and the MMS.

The study constituted a Ministry of Health initiated data quality evaluation and is approved by the Ministry of Health.

## Results

Table 4 presents the average IRR scores for the 24 indicators, the two indicator categories, the five domains, and overall scores from each of the three IRR assessments. The IRR scores for the rational drug use sub-indicators are presented in Additional file 4.

**Table 4:** Average IRR scores (%) for 24 SPARS indicators and tests of change, by domain and indicator category

		Assessment		Assessments		
	1	2	3	1 to 2	2 to 3	1 to 3
Number of teams (facilities assessed by each team)	2 (3)	10 (2)	10 (2)			
Number of assessments	n=6	n=20	n=20	iwo sample test lor proportions		
Dispensing quality domain						
1. Dispensing time	67	55	60	0.602	0.749	0.757
2. Packaging material	83	100	100	0.060	-	0.060
3. Dispensing equipment	71	90	80	0.245	0.376	0.641
4. Services available at dispensing area	67	81	78	0.470	0.814	0.583
5. Patient care	37	72	64	0.117	0.588	0.240
6. Labeling	79	83	75	0.823	0.535	0.841
7. No discrepancy between prescribed and dispensed medicines cotrimoxazole/ amoxicillin- Rational prescribing	50	45	75	0.829	0.053	0.245
Dispensing quality domain	**65**	**75**	**76**	**0.630**	**0.941**	**0.593**
Prescribing quality domain						
8. Correct use of prescription recording system	33	65	70	0.164	0.736	0.102
9. Rational Prescribing	30	76	63	0.038	0.372	0.154
10. Adherence to standard treatment guidelines diarrhea	67	60	60	0.757	1.000	0.310
11. Adherence to standard treatment guidelines cough and cold	67	45	65	0.345	0.204	0.928
12. Adherence to standard treatment guidelines malaria	25	65	63	0.084	0.895	0.101
Prescribing quality domain	**44**	**62**	**64**	**0.434**	**0.896**	**0.382**
Sto ck management do main						
13. Availability of stock card/ledger book	17	55	85	0.102	0.038	0.002
14. Correct filling of stock card	50	50	55	1.000	0.752	0.829
15. Does physical count agree with stock card	100	75	90	0.173	0.212	0.420
16. Stock book correctly filled	100	95	75	0.576	0.077	0.173
Sto ck management do main	**67**	**69**	**76**	**0.926**	**0.62**	**0.66**
Storage management domain						
17. Cleanliness of the pharmacy	33	40	55	0.757	0.342	0.345
18. Hygiene of the pharmacy	57	77	75	0.337	0.882	0.395
19. System of storage of medicines and supplies	63	84	79	0.267	0.684	0.425
20. Storage conditions	79	88	88	0.578	1.000	0.578
21. Storage practices of medicines in pharmacy (stores and dispensary)	64	77	68	0.524	0.524	0.855
Storage management domain	**59**	**73**	**73**	**0.513**	**1.00**	**0.513**
Ordering and reporting domain						
22. Reorder level calculation	33	50	95	0.464	0.001	0.001
24. Accuracy of HMIS report	67	70	45	0.889	0.110	0.345
25. Filing	50	45	70	0.829	0.110	0.366
Ordering and reporting domain	**50**	**55**	**70**	**0.829**	**0.327**	**0.366**
Overall Score	**57**	**67**	**72**	**0.653**	**0.731**	**0.488**
Indicator categories						
Complex	**55**	**65**	**68**	**0.657**	**0.841**	**0.558**
**Simple**	**60**	**71**	**75**	**0.611**	**0.776**	**0.475**

### I. Overall IRR score (all 24 indicators)

The overall IRR score across all indicators improved from 57% in 2011 to 72% in 2013. The number of indicators with an acceptable IRR score (≥75%) increased from five indicators (21% of the 24 indicators) in 2011 to 12 indicators (50% of the indicators) in 2013.

**Fig. 2:**
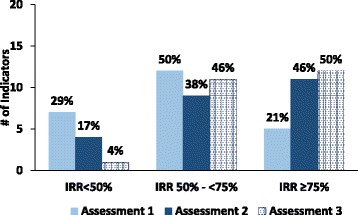
Distribution of indicators by IRR score, at first, second, and third assessments

**Fig. 3:**
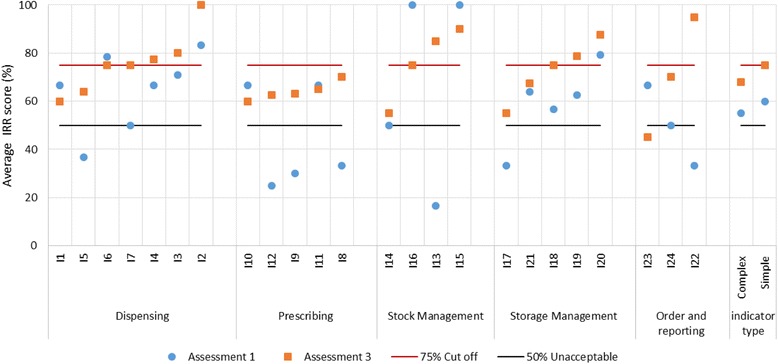
Inter-rater reliability scores for 24 SPARS indicators and complex & simple indicator types at first and third assessments for 2011–2013. *Optimal IRR score is 100%, acceptable score of ≥75% marked with red line and 50% marked with black line

Of the 24 indicators, the IRR scores of 17 (71%) improved between the initial and third assessments. The average IRR indicator improvement for the 17 indicators was 24 percentage points (range: 4%–68%). The IRR scores for seven indicators got worse between the first and third assessment with an average reduction of 11% points (range: − 2% to − 25%) (Table 4). The number of indicators with unacceptable IRR scores (i.e., IRR < 50%) fell from seven to only one following the two interventions (Fig. 2). Figure 3 depicts the IRR scores for each indicator presented by domains at the first and third assessments. Between the first and third assessment the IRR score ranges narrowed with more indicators having an IRR score of 75%, indicating improvement in reproducibility and data quality over the study period from 2011 to 2013.

### II. IRR scores for indicator and domains

#### Dispensing quality

Two indicators in this domain, *packaging material* and *labelling*, had an acceptable IRR score of ≥75% at all three assessments. Three additional indicators had an acceptable reproducibility score at the third intervention (not significant improvement): *dispensing equipment*, *services available at dispensing area,* and *no discrepancy between prescribed and dispensed medicine—cotrimoxazole/amoxicillin.* Two indicators, *dispensing time* and *patient care*, were below the acceptable reproducibility score by more than 10 percentage points at the final assessment. The overall IRR score for the dispensing domain at the third assessment was acceptable (76%).

#### Prescribing quality

Of the five indicators in this domain, only one*, rational prescribing*, had acceptable reproducibility at only the second assessment, after improving significantly from the first to second assessment (*p* = 0.038). Three indicators, *correct use of recording system, rational prescribing,* and *adherence to treatment guidelines for malaria*, improved considerably between the first and third assessments (not significant), but did not reach the acceptable reproducibility benchmark. Overall, the prescribing quality domain IRR score improved but remained not acceptable at the final assessment (64%).

#### Stock management

Three of the four indicators, *availability of stock card*, *agreement between stock card and physical count* and *stock book filled correctly,* had acceptable reproducibility scores at the third assessments, although the IRR for the latter two declined over time (not significant)*.* One indicator, *availability of stock card*, improved significantly from the first to the third assessment (*p* = 0.002). *Correct filling of stock card* continued to be difficult to assess in a unified manner and had a low IRR score despite our efforts to improve IRR. Overall, however, the stock management domain IRR score was acceptable at the final assessment (76%).

#### Storage management

One indicator in this domain, *storage conditions*, had an acceptable reproducibility score at all three assessments. By the third assessment, two other indicators also achieved an acceptable reproducibility score: *hygiene of the pharmacy* and *systems of storage*. One indicator, *cleanliness of the pharmacy,* had the largest improvement between the first and third assessments (not significant) but with an IRR score far below the acceptable reproducibility threshold of ≥75%. The overall IRR score for the storage domain remained just below the acceptable threshold (73%).

#### Ordering and reporting

Of the three indicators in this domain, one—*reorder level calculation—*achieved an acceptable IRR score by the third assessment with a significant improvement between the first and third assessment (*p* = 0.001). *Accuracy of the health management information system (HMIS) report* declined from moderately acceptable reproducibility to unacceptable (not significant). The domain IRR score improved following the interventions, but remained just below the acceptable threshold of ≥75% (70%).

### III. IRR scores for simple or complex indicator categories

In the initial assessment, neither of the two indicator categories, simple or complex, had an acceptable IRR score. IRR scores improved following two assessments for both categories, with the simple indicators improving by 15 percentage points between the initial and third assessments (*p* = 0.475) and complex indicators improving by 13 percentage points (*p* = 0.558).

The complex category did not reach the ≥75% threshold of acceptable reproducibility by the third assessment, however, the simple indicator category just reached the 75% cut-off (Fig. 3).

### IV. IRR scores and rater (MMS) team characteristics

We found no statistically significant relationship between the IRR scores and any of the characteristics of the MMS raters—gender, profession (e.g., clinical officer), or average number of prior SPARS supervisory visits completed (Additional file 5).

## Discussion

This study measured IRR for the 24 SPARS medicines management indicators used to assess performance in the Ugandan pharmaceutical sector at three different time points and examined whether IRR increased after efforts to improve reproducibility. The SPARS data collection tool uses well-known indicators, the tool was thoroughly piloted, and the MMS received three weeks of combined classroom and practical training. Despite this preparation, we found that initially the MMS’ IRR scores for the medicines management indicators in the SPARS assessment was poor; only five of the 24 indicators achieved an acceptable IRR of ≥75%. Our findings highlight the fact that IRR must be considered when designing indicator-based assessments, even when using well-known and globally recognized indicators and extensively trained data collectors.

Pharmaceutical sector indicators that assess rational drug use and supply chain performance are used to guide policies and system change [10, 11, 14]. These indicators are widely accepted as an objective and standard measure of rational use of medicines and medicines management and have been used in more than 30 mainly developing countries [21]. However, very few programs make the effort to assess the temporal and inter-rater reliability of the indicators they use. Therefore, little is known about the reproducibility of pharmaceutical sector indicators [13]. A systematic literature review of the use of medicine-related indicators in Southeast Asia found little information on validity, reliability, and feasibility of these indicators, especially those not promoted by World Health Organization [13]. The World Health Organization drug use indicators have been developed using appropriate methods, tested in numerous countries, applied in a standardized way in many studies and are widely accepted [10, 13, 22, 23]. Nevertheless, we found that both World Health Organization and non- World Health Organization indicators had poor inter-rater reliability. Our study is one of the first that measures and documents the IRR of pharmaceutical and rational drug use indicators. Moreover, we suggest multi-pronged interventions to increase IRR of problematic pharmaceutical sector indicators.

This study suggests that focused and practical training and tailored instructions may improve IRR scores for pharmaceutical and medicines management indicators; at the final assessment half (12) of the 24 indicators achieved an acceptable ≥75% IRR and only one had an IRR of less than 50%. IRR of indicators in all five domains improved following the interventions, reaching acceptable or almost acceptable scores; only the prescribing quality domain indicators continued to have low IRR. Both the IRR and the effectiveness of our efforts to improve IRR depended greatly on the type of indicator. Not surprisingly, indicators that involve complex calculations, detailed sampling, and a high degree of judgement required the most effort to achieve acceptable reliability. Prescribing domain indicators are all complex indicators and have very specific data collection methods. The MMS were trained in sampling and the complicated calculations; however, correctly assessing these complex indicators remained a challenge for many MMS, even with extra attention. Therefore, although the domain had the highest improvement in IRR score overall, it remained the domain with the lowest reproducibility in scores.

Despite overall improvement, the IRR score did not improve for all SPARS indicators. IRR for two indicators decreased by over 20 percentage points: *stock book is filled in correctly* and *accuracy of the HMIS report*. At the time of the first assessment in 2011, most facilities had not yet received the new stock book, and the indicator could therefore only be scored “not applicable.” Later when all the facilities received stock books, the MMS needed to know how to fill in the stock book correctly to assess the indicator, causing reproducibility to decline. Each facility reports data on a monthly basis into a centralized HMIS. The HMIS report includes data on availability of a selected basket of medicines and health supplies and patient attendance figures. A new HMIS form was introduced to the health facilities at the end of 2012. Assessing accuracy of the data reported in the new HMIS form is likely to have declined due to the introduction of the new HMIS form without related training for the MMS.

This study has multiple limitations. Although the overall IRR score increased over the three assessments, we cannot attribute the improvement to the revised tool and training because we did not use a controlled design; other changes, such as MMS gaining more experience over time, changes in the MMS used as raters, or changes in the sample of facilities assessed could have contributed to the improvement in the IRR scores. MMS experience increased across assessments as they made more visits, and the threshold for MMS to be considered experienced increased from ≥7 SPARS facility visits initially to ≥12 SPARS visits in the last two assessments. Though the composition of the assessment team remained consistent with one less experienced and two experienced MMS’s, the number of visits that comprised the definition of “experienced” rose after the first assessment; therefore, the teams became more experienced overall. However, because we observed improvements in both the second and third assessment with same threshold for experience (≥12), the revised tool and training likely contributed to reproducibility improvements.

The study is also limited by the small number of observations in the initial assessment, which resulted in insufficient power to detect statistically significant differences between the three assessments. We included the initial findings because they demonstrated the need to improve IRR. We limited the later assessments to lower level facilities because they manage fewer pharmaceutical products than higher level facilities and have only one medicines storage area, which shortens the time MMS need to collect the SPARS data and allows more time for supportive supervision; lower level facilities also constitute 93% of all public sector health facilities.

We chose to measure IRR using percentage agreement instead of Fleiss kappa coefficient, which measures inter-rater agreement among three raters, because we did not have a sufficient number of facilities per MMS team to calculate kappa [18, 24]. Compared to other IRR methods, the percentage agreement approach tends to overestimate IRR due to chance agreement. However, our method was conservative, requiring agreement among three raters instead of the more commonly used agreement between two raters. Finally, we did not assess the validity of the indicators because we did not have a gold standard.

Measuring performance using the SPARS indicators has been proven to be feasible and useful to identify medicines management problems and to track the impact of SPARS in health facilities in Uganda. Uganda now has in place a strong capacity building strategy with indicators, training approaches, and data collection methods that ensure reproducible results for most of the indicators, not only for guiding the supervision and tracking improvements, but also for informing national pharmaceutical policy.

## Conclusion

Health program managers must have access to reliable information to identify problems, monitor progress accurately, and make evidence-based decisions. Often such information is obtained through indicator-based tools, but the reliability of these indicators is unknown. By testing the IRR of the SPARS assessment indicators, we identified problems in how supervisors understood and calculated the indicators. Our study suggests that targeted and multi-pronged efforts including training, tool revisions, and repeated instructions can improve reproducibility of the SPARS indicator ratings. We now have a set of indicators with an average IRR score of 72%, just shy of the acceptable level, and three of five domains that achieved an acceptable IRR of ≥75%. We learned that, where possible, it is best to use simple binary indicators when designing an indicator-based assessment tool and that assessing and improving IRR should be an iterative process. Having uniform data reproducibility standards, assessment methods, and guidelines for best practices to evaluate IRR of indicators would make it easier for more programs in resource-limited countries to improve their data quality.

## Additional files

Additional file 1: SPARS indicator based data collection and performance assessment tool dated January 2013. (PDF 964 kb)

Additional file 2: The final SPARS data collection guidelines October 2011. (PDF 246 kb)

Additional file 3: Illustrative example of IRR score calculation for SPARS indicators, sub-indicators, and domains across the rater teams. (PDF 99 kb)

Additional file 4: IRR score for the rational drug use sub indicators assessments 1–3. (PDF 502 kb)

Additional file 5: Relationship assessment using logistic regression between MMS group characteristics and domain score measures by IRR scores ≥75% categorized as acceptable. (PDF 321 kb)

## Abbreviations

HMIS: Health management information system
IRR: Inter-rater reliability
MMS: Medicines management supervisors
SPARS: Supervision performance assessment and recognition strategy
