# Supplementary material for: Evaluating inter-rater reliability of indicators to assess performance of medicines management in health facilities in Uganda
Source: J Pharm Policy Pract. 2018 May 3;11:11. doi: 10.1186/s40545-018-0137-y (PMC5932790; doi:10.1186/s40545-018-0137-y)
Supplement: Supplementary file 2 — The final SPARS data collection guidelines October 2011. (PDF 246 kb) [file 40545_2018_137_MOESM2_ESM.pdf]

KEY POINTS TO NOTE WHEN USING THE SUPERVISION MONITORING AND REPORTING TOOL (ROUTINE TOOL)

This is a short guideline for filling out the health facility supervisor's monitoring and reporting tool. All Medicine Management Supervisors (MMS) must read this guideline before starting supervision of health facilities.

NA/0/blank field

When filling out the routine supervision tool, never leave any fields blank. In case there is no data for a field, it must be filled with NA or data must be found. NA is used when the data is not applicable meaning that the data cannot be found. For example order dates for HC2s and HC3s that receive kit and does not order or in case there is no stock card and therefore you cannot answer questions where the stock card has the information. 0 can never be used in cases where you have no data, because 0 means that your answer is 'no' or the number 0. Whenever you use NA, please make a comment explaining why you could not obtain the data. IF you cannot get data and the field is applicable then the field has to be blank. Blank fields are normally used if the data collector/supervisor forgot to get data. If you leave a field blank, explanation is required or the MMS can be asked to go back to the facility to get the data.

Dispensing time (Indicator no. 1)

When measuring the time, do not include the time for getting the prescription and medicine; only measure the time when the dispenser gives information to the patient. If any interruption is made while the dispenser is giving information, stop the clock. Resume counting when the dispenser is giving information again, and add up the total time

Packaging material (Indicator no. 2)

Appropriate packaging is dispensing envelope for tablets/capsules and clean, not reused bottle specifically for dispensing liquids from JMS or locally procured.

Dispensing equipment (Indicator no. 3)

Graduated measuring cylinder must be 1/0 – not NA – like for the other equipment.

Services available at the dispensing area (Indicator no. 4)

Privacy is observed when the other patients are at least 2 metres away from the dispenser and patient, if the dispenser speaks softly so other patients can't hear the conversation or if there is a clearly marked line on the floor indicating how far away the patients should be (and it is adhered to). Hand washing facilities for patients should be found within the facility and should include running water and soap. Drinking water must be in the dispensary and should be water which is drinkable for example stored in a jerry can.

Patient care and labelling (Indicator no. 5 & 6)

Fill in these 2 indicators together. After checking number of medicine prescribed and dispensed select one of the medicines the patient has received to fill out the rest of table 5 and the entire table 6. If no medicine was dispensed then choose another patient who received medicine. When calculating the percentage scores for patient care, use the method (sum/5)*100 for the 4 columns indicated except for discrepancy where you use the method 100 – [(sum/5)*100]. This method gives the answer for

percentage non-discrepancy which we are looking for. Medicine name should be generic name and brand name. Generic name can be an abbreviation.

Non-discrepancy between prescribed and dispensed medicines (antibiotics) (Indicator no. 7)
The score is non-discrepancy like for indicator 5, so use this formula 100 – [(sum/5)*100]. Write in comments if there has been stock out or if anything else had influence on the result.

Correct use of prescription recording system (Indicator no. 8)
Only use 1/0 – never write the date, patient number, diagnosis etc. When assessing name of medicine it must be generic name or brand name and generic name. Generic name can be abbreviated.

Rational prescribing (Indicator no. 9)
When filling in the table, remember that diagnosis is definite diagnosis e.g. malaria, URTI, dysentery NOT symptoms e.g. fever, chest pain, back ache, etc. Sample by counting the number of pages used over the 2 months, then divide by 20 to get the sample interval. If there is 60 pages, then use the prescription on top of every 3rd page (60/20). There are many other indicators under rational prescribing; these should be calculated as follows:

- Average no. of medicines prescribed per patient: divide the total no. of medicines e.g. 67 by the number of prescriptions sampled, 20. Write this figure to one decimal place, e.g. 67/20 = 3.4
- % of medicines prescribed by generic name: add up the medicines prescribed by generic name and fill in the total e.g. 45 (out of the total no. of medicines, e.g. 67). % is calculated as 45/67*100=67%. Write as a whole number, no decimals.
- % of patients prescribed 1 or more antibiotics: Count the number of patients receiving antibiotics (e.g. 13) out of the total prescriptions sampled (20). % is calculated as: 13/20*100=65%, as a whole number.
- % of drugs being antibiotics: Add up the drugs that are antibiotics (e.g. 20) out of the total number of drugs (67) prescribed. % is calculated as 20/67*100=30%, as a whole number
- % of patients prescribed 1 or more injections: Count the number of patients receiving injections (e.g. 3) out of the total prescriptions sampled (20). % is calculated as: 3/20*100=15%, as a whole number.
- % diagnosis recorded: Count the number of '1's (e.g. 17) and divide by total number of prescriptions sampled (20) i.e. 17/20*100=85%, as a whole number.

Diarrhoea (No blood) (Indicator no. 10)
The appropriate diagnosis is diarrhoea. Only look at prescriptions for the last month or last supervision date from date of supervision visit.

Cough/cold (Indicator no. 11)
The appropriate diagnosis for this are: Non-pneumonia, ARI (acute respiratory infection), ARTI (acute respiratory tract infection), common cold, flu, cough, cold, sore throat. Only look at prescriptions for the last month or last supervision date from date of supervision visit.

Malaria treatment (Indicator no. 12)
A functional lab is one with a lab technician and all the required reagents to perform a test. If there is no functional lab or Rapid Diagnostic Tests (RDT) (i.e. if the response for both = 0), write 'NA' when inquiring where a test was conducted.

Availability and correct use of stock card and stock book (Indicator no. 13-16)
This indicator is divided up into 4 indicators that need to be calculated. These are made in bold in the table. 'Stock card availability (indicator no. 13)', 'Correct filling of stock card (14)', 'Does physical count agree with stock card balance (15)' and 'Is stock book correctly used(16)?' For all these indicators, get

the sum of yes (1) and divide by the total number of medicines minus any 'Not applicable' (NA) (total is 15, if all medicines are stocked in the facility).

Correctly filled stock card has all fields in the header filled, which is normally name, strength, formulation, storage conditions, tin size, AMC and min/max stock level.

Physical count must be done every month and can be marked in another book or on the stock card, score 1. But encourage that it is written in stock card in future. If not marked in stock card, score 0.

Use the date of survey/supervision visit for calculations when looking at a time period. Only exceptions are for calculation of issued out and stock out days in last 3 months if item was stocked out for 3 months. In that case, use the latest 3 months with stock available to enable calculation of AMC.

Never leave any fields blank and differentiate between NA and 0. Always have an explanation for NA and blank fields – for more information on NA and 0, see beginning of this guideline.

When finishing the exercise, the stock cards and stock should be left in the correct way as part of the supervision and the store keeper has to be informed about the changes.

Cleanliness of the pharmacy (indicator 17)
Assessment of the cleanliness is based on the order in which the medicine is stored (even though boxes are on floor they must be stored in a tidy way) and the amount of dirt and dust.

Hygiene of the pharmacy (indicator 18)
Toilet and hand washing facilities are acceptable, hygienic and functioning if the MMS would use the facilities. Toilet paper and soap can be kept inside the facility but it should be available to all staffs.

System for storage of medicines and supplies (indicator 19)
The system for storage can be alphabetical, therapeutic or according to formulation. Labelling of the shelves must be clear to read.

Storage conditions (indicator 20)
For storage conditions use the following guidelines;

a. Sunlight cannot enter at all and if window painted it must not be in a dark colour.
b. A window that can be opened is not enough for ventilation, a ventilator must be available.
c. Adequate store space is when medicine can be stored correct and it is possible to move freely around the store.
d. Sand is acceptable as fire safety equipment, but there must be enough sand and it must be possible to throw it on the fire.
e. Vaccines must be in the middle of the fridge, it is not enough that they are not in the side of the fridge.
f. If there is more than one fridge in the facility, use the UNEPI fridge for assessment.

Storage practices of medicines in the pharmacy (Indicator no. 21)
Though there are no expired medicines at the day of survey, b) and c) can still be assessed. The facility must have a record and space for storage of expired medicine in case necessary.

Reorder level calculation (Indicator no. 22)
It is important to write the results of calculation of quantity to order to show that staff was capable. Understanding the VEN concept applies to all including HC 2 and HC 3 even though they receive kit.

Timeliness of orders and distribution (Indicator no. 23)
Complete the dates from the most recent order. The score needed is for order timeliness only. Fill in all the dates for rows 1 to 5 for HC IVs and hospitals; only fill in rows 3 to 5 for HC IIs and IIIs as these do not make orders. Fill NA in the first two rows for orders. The scheduled dates are available from the NMS order and delivery schedule. Use the calculation as indicated in the tool.

Accuracy of HMIS report (Indicator no. 24)
Mark (1/0) if the stock out days for each item was available in the latest HMIS 105 report and then write number of days out of stock according to report and compare with stock out days according to stock card for that month.

Filing (Indicator no. 25)
Orders are scored NA for HC 2 and HC 3.

Lead time (Indicator no. 26)
Pay attention to the dates written on the order form, delivery note and invoice.

- Date of order stamp: This is the date of the stamp or signature by the facility when the order was made
- Date of order approval: This is the date of the stamp or signature of approval by the DHO
- Date order received at NMS: This is the date the order was received at NMS; it may not be easy to obtain if the order was not hand delivered. If you can't get it, write 'Not available'.
- Date order completed at NMS: Fill in the date of the NMS stamp on the delivery note
- Date supplies received at district: This is the date of the stamp/signature that the district stores assistant received the supplies
- Date supplies received at the facility: This is the date the in-charge of the facility signed for receipt of the supplies

Not applicable for HC2s and HC3s. Write NA for all and write comment below. If order and delivery notes are not available or cannot be paired write NA and make a comment below.

Accepted abbreviations

AL	Arthemether and Lumefantrine
AS/AQ	Artesunate/Amodiaquine
ASA	Acetylsalicylic Acid
CAF	Chloramphenicol
CQ	Chloroquine
CTX	Cotrimoxazole
FEFOL	Ferrous Sulphate and Folic Acid
ORS	Oral Rehydration Salt
PCM	Paracetamol
QNN	Quinine
SP	Sulphadoxine and Pyrimethamine

Generic names for commonly prescribed medicines and examples of brand names

Generic name	Examples of brands
**Antibiotics**	
1. Amoxicillin 250mg capsules	Amoxyl, Paromyx
2. Cotrimoxazole 480mg tab	Septrin, Trimago, Bactrim, Cotrimox, Kamcotri
3. Ciprofloxacin 500mg tab	Ciprobid
4. Doxycycline 100mg capsules	
5. Benzyl penicillin IMU injection	X-pen
6. Chloramphenicol 1g injection	
7. Gentamycin 80mg/2ml injection	
**Antimalarials**	
8. Artemether/Lumefantrine	Coartem, Lumefantrine
9. Sulfadoxine/Pyrimethamine (SP) 500/25mg tab	Fansidar, Kamsidar
10. Quinine sulphate 300mg tab; Quinine dihydrochloride 300mg/ml injection	

**Analgesics**	
11. Paracetamol 500mg tab	Panadol, Kamadol, Renedol
12. Ibuprofen 200mg tab	Brufen
13. Diclofenac 50mg tab	Olfen, Diclodenk
14. Indomethacin 50mg capsule	Indocid, Indocil
15. Acetyl salicylic acid (ASA) 300mg tab	Aspirin
**Antamoebic medicines**	
16. Metronidazole 200mg tabs	Flagyl, Metrogyl
**Anti histamines**	
17. Chlopheniramine 4mg	Piriton
18. Cetrizine 10mg	
**Others**	
19. Diazepam 5mg tab or 10ml injection	Valium
20. Chlorpromazine 25 or 100mg tab	Largactil
21. Bendrofluazide 5mg tab	Aprinox
22. Propranolol 40mg tab	Inderal
23. Mebendazole 100mg tab	Vermox
24. Albendazole 200 or 400mg tab	Alben
