# Supplementary material for: Evaluating inter-rater reliability of indicators to assess performance of medicines management in health facilities in Uganda
Source: J Pharm Policy Pract. 2018 May 3;11:11. doi: 10.1186/s40545-018-0137-y (PMC5932790; doi:10.1186/s40545-018-0137-y)
Supplement: Supplementary file 3 — Illustrative example of IRR score calculation for SPARS indicators, sub-indicators, and domains across the rater teams. (PDF 99 kb) [file 40545_2018_137_MOESM3_ESM.pdf]

Illustrative example of IRR score calculation for SPARS indicators, sub-indicators, and domains across the rater teams

Indicator	Rater team 1 agreement (%)	Rater team 2 agreement (%)	Rater team 3 agreement (%)	Rater team 4 agreement (%)	Indicator IRR score (%)
1. Dispensing time	0	100	0	100	50
2. Packaging material	100	0	100	100	75
3. Dispensing equipment	100	100	100	100	100
**Dispensing quality domain**					**75**
9. Rational prescribing (with 5 sub-indicators)					
Average # of medicines prescribed per patient	0	100	0	100	50
% of medicines prescribed by generic name	100	0	100	100	75
% of patients prescribed 1 or more antibiotics	100	100	100	100	100
% of patients prescribed 1 or more injections	0	100	0	100	50
% diagnosis recorded	0	0	0	0	0
**9. Overall Indicator score (with sub-indicators)**					**55**
