# Supplementary material for: Evaluating inter-rater reliability of indicators to assess performance of medicines management in health facilities in Uganda
Source: J Pharm Policy Pract. 2018 May 3;11:11. doi: 10.1186/s40545-018-0137-y (PMC5932790; doi:10.1186/s40545-018-0137-y)
Supplement: Supplementary file 4 — IRR score for the rational drug use sub indicators assessments 1–3. (PDF 502 kb) [file 40545_2018_137_MOESM4_ESM.pdf]

Mean IRR score for the SPARS rational drug use sub indicators assessment 1-3

	Assessment			Assessments		
	1	2	3	1 to 2	2 to 3	1 to 3
**Number of teams (facilities assessed by each tean**	**2 (3)**	**10 (2)**	**10 (2)**	**Two sample test for proportions**		
**Number of assessments**	**n=6**	**n=20**	**n=20**			
**Rational prescribing**						
Number of medicines prescribed	33.3	85.0	80.0	0.012	0.677	0.029
Number of medicines prescribed by generic name	16.7	65.0	40.0	0.039	0.113	0.299
Patients receiving one or more antibiotics	0.0	65.0	50.0	0.005	0.337	0.027
Patients receiving one ore more injections	66.7	90.0	75.0	0.170	0.212	0.698
Patients with a diagnosis recorded	33.3	75.0	70.0	0.058	0.723	0.102
**9. Rational Prescribing**	**30**	**76**	**63**	**0.038**	**0.372**	**0.154**
