# Supplementary material for: Evaluating inter-rater reliability of indicators to assess performance of medicines management in health facilities in Uganda
Source: J Pharm Policy Pract. 2018 May 3;11:11. doi: 10.1186/s40545-018-0137-y (PMC5932790; doi:10.1186/s40545-018-0137-y)
Supplement: Supplementary file 5 — Relationship assessment using logistic regression between MMS group characteristics and domain score measures by IRR scores ≥75% categorized as acceptable. (PDF 321 kb) [file 40545_2018_137_MOESM5_ESM.pdf]

Relationship between MMS group characteristics and domain IRR scores >75%

MMS Group composition	Dispensing			Prescribing			Stock Management			Storage Management			Ordering and reporting			Total score		
	n/N*	%	OR (95%CI)	n/N	%	OR (95%CI)	n/N	%	OR (95%CI)	n/N	%	OR (95%CI)	n/N	%	OR (95%CI)	n/N	%	OR (95%CI)
**Total**	**24/46**	**52**		**15/46**	**33**		**11/46**	**24**		**27/46**	**59**		**15/46**	**33**		**17/46**	**37**	
**Gender**																		
Three Males	12/22	55	1.00	8/22	36	1.00	7/22	32	1.00	11/22	50	1.00	8/22	36	1.00	8/22	36	1.00
Two Males	9/18	50	0.83 (0.24-2.90)	5/18	28	0.67 (0.17-2.59)	3/18	17	0.43 (0.09-1.98)	12/18	67	2.00 (0.55-7.25)	5/18	28	0.67 (0.17-2.59)	6/18	33	0.88 (0.24-3.24)
One Male	3/6	50	0.83 (0.14-5.08)	2/6	33	0.88 (0.13-5.89)	1/6	17	0.43 (0.04-4.39)	4/6	67	2.00 (0.30-13.26)	2/6	33	0.88 (0.13-5.89)	3/6	50	1.75 (0.28-10.81)
**Profession**																		
Zero/one Clinician	8/19	42	1.00	6/19	32	1.00	5/19	26	1.00	11/19	58	1.00	6/19	32	1.00	8/19	42	1.00
Two Clinicians	9/17	53	1.55 (0.41-5.78)	5/17	29	0.90 (0.22-3.74)	4/17	24	0.86 (0.19-3.92)	10/17	59	1.04 (0.28-3.92)	6/17	35	1.18 (0.30-4.73)	6/17	35	0.75 (0.19-2.89)
Three Clinicians	7/10	70	3.21 (0.63-16.38)	4/10	40	1.44 (0.29-7.10)	2/10	20	0.70 (0.11-4.48)	6/10	60	1.09 (0.23-5.18)	3/10	30	0.93 (0.18-4.90)	3/10	30	0.59 (0.12-3.01)
**Average number of visits**																		
<10	8/14	57	1.00	5/14	36	1.00	4/14	29	1.00	7/14	50	1.00	5/14	36	1.00	7/14	50	1.00
10-19	4/8	50	0.75 (0.13-4.29)	2/8	25	0.60 (0.09-4.17)	0/8	0	-	8/8	100	-	1/8	13	0.26 (0.02-2.73)	1/8	13	0.14 (0.01-1.49)
20+	12/24	50	0.75 (0.20-2.83)	8/24	33	0.90 (0.3.88-1.43)	7/24	29	1.03 (0.24-4.41)	12/24	50	1.00 (0.27-3.74)	9/24	38	1.08 (0.27-4.25)	9/24	38	0.60 (0.16-2.28)

*n/N Number of assessments with a score of >75%/Total number of assessments
